# Supplementary material for: The influence of proximity and knowledge base on recombination innovation in R&D collaboration
Source: PLoS One. 2024 Feb 27;19(2):e0298735. doi: 10.1371/journal.pone.0298735 (PMC10898747; doi:10.1371/journal.pone.0298735)
Supplement: S1 Appendix — (DOCX) [file pone.0298735.s001.docx]

**S1 Appendix. Calculation of the geographic distance between two cities.**

The geographic distance between the two cities is calculated based on the formulation below, using the coordinates of the city.

$$d=6357*arccos[\sin\left( \mathrm{wA} \right)\sin\left( \mathrm{wB} \right)+\cos\left( \mathrm{wA} \right)\cos\left( \mathrm{wB} \right)*\cos\left( jA-jB \right)]$$

where wA and wB is the latitude of city A and B respectively, and jA and jB is the longitude of the city A and B. 6357 is the polar radius. We can obtain the geographical distance in kilometre using this formula.
