# Supplementary material for: The influence of proximity and knowledge base on recombination innovation in R&D collaboration
Source: PLoS One. 2024 Feb 27;19(2):e0298735. doi: 10.1371/journal.pone.0298735 (PMC10898747; doi:10.1371/journal.pone.0298735)
Supplement: Attachment — Submitted filename: Response letter.docx [file pone.0298735.s002.docx]

**RESPONSE LETTER: PONE-D-22-33904**

*Dear editors and reviewers,*

*Thank you for giving us the opportunity to submit a revised draft of our manuscript titled “The influence of proximity and knowledge base on recombination innovation in R&D Collaboration” to Plos One. We appreciate the time and effort you and the reviewers have dedicated to providing your valuable feedback on our manuscript. We are grateful to the reviewers for their insightful comments on our paper.*

*We have been able to incorporate changes to reflect most of the suggestions provided by the reviewers. We have taken your advice seriously and framed the manuscript. Below you can find a point-by-point response to your comments. Again, we thank you wholeheartedly for allowing us to resubmit a major revision of our study.*

**Reviewer 1 comments：**

In their paper, the authors investigate the effects of proximity (geographical and technical) on the recombination innovation at the dyad level between companies. Additionally, the authors test how this effect is mediated by an organization's knowledge base structure. Thus, based on theories from the R&D field, the authors propose a theoretical framework that explains the interactive effect of proximity and knowledge network structure on companies' collaborative recombination innovation potential. They derive six hypotheses that test for the direct and interactional effects of the aforementioned variables. For their empirical evaluation, the authors utilize longitudinal patent data from 150 organizations in the global nanotechnology industry. After conducting their statistical analyses, the authors conclude that technological proximity exerts a negative effect on the recombination innovation of companies while geographic proximity has an inverted U-shaped effect. Moreover, they were able to show that an organization's knowledge base decomposability plays a negative role in moderating the effect of technological proximity while playing a positive role in regulating the effect of geographic proximity. Degree centrality on the other side positively moderates the effect of both technological and geographic proximity.

In this review, I will highlight the research’s strengths and weaknesses and outline the challenges and opportunities presented in the manuscript.

I enjoyed reading the manuscript and appreciate the authors’ work. They succeed in tackling an interesting subject by developing a comprehensive theoretical framework and conducting thorough empirical analyses. By discussing different potential effects of the indicators under scrutiny, the authors derive six plausible hypotheses and test them convincingly in their empirical analyses. Additionally, the included figures and tables are clearly arranged and help the reader to follow their conceptual as well as empirical procedure. Overall, the authors use reasonable language, which makes it possible to follow their presented ideas most of the time.

While the paper succeeds in delivering its main messages, my review reveals potential issues that might diminish the contribution of the presented manuscript:

1. No direct research question

In their introduction, the authors mention that they aim to analyze "how knowledge interdependence at the micro level and partners’ knowledge origin interact leading to the change in state of collaborative recombination innovation." Although they mention their research goal, it would be helpful if they could include precise research questions which would make it easier for the reader to understand the overall goal of the paper.

*Thank you for your constructive and useful review. The motivation of the study is unclear in the previous version of the paper. In the revised version, we rewrote the Introduction part to illustrate the research question of the study more clearly.*

*We put forward the practical question “there are a large number of cases of dissolution and failure of inter-organizational collaborations. The high failure rate of inter-organizational collaborations restricts the development of firms’ recombination innovation.”*

*Then, we also put forward the research question “How partner selection affect firms’ collaborative recombination innovation?” and also briefly introduce the main analytical lens of this paper as “this paper attempts to explore the characteristics of collaborative partners that influence firms’ collaborative recombination innovation based on the proximity perspective as well as the characteristics of organizations’ knowledge base”.*

2. Introduction of theories

When introducing their concept of proximity, the authors mention the analytical framework of Boschma with its five dimensions of proximity. Yet, they never explain those five dimensions in detail. To help the reader understand their theoretical framework more thoroughly, the authors should introduce their utilized theories in more detail.

*Thank you for your constructive and useful review. As you suggested, we added explanations about the Boschma’s analytical framework of five dimensions of proximity on Page 6.*

3. Operationalization of variables

It has to be positively emphasized that the authors operationalize all mentioned variables by including a measurement description thereof. Yet, in several cases (e.g. technological proximity or control variables like structural hole) the authors do not explain how the measurement relates to the theoretical concept. It would be helpful if the authors could highlight the link between concept and measurement more thoroughly. When mentioning their formula to calculate the spatial distance, the authors do not explain the meaning of the number "6357." Please explain this formula in more detail. Additionally, if the authors establish a link between concept and measurement (e.g. knowledge base decomposability), the paper could be enhanced if the authors could provide more information regarding the theoretical background (e.g. what is the four-digital level class of IPC?).

*Thank you for raising this important point. As you suggested, we added explanations about the independent and control variables in detail on Page 20, 22, and 23. In this way, we explain the link between concept and measurement more thoroughly.*

*As suggested by the reviewer, we moved the description on the measurement of geographic distance to the Appendix. We explained the meaning of 6357 as “6357 is the polar radius” on Page 39.*

*We gave theoretical background of technological classes on Page 19 and also explained the meaning of the four-digit level IPC class on Page 22. We also give the theoretical background of other main variables such as degree centrality and recombination innovation.*

4. Creation of dataset

When curating their dataset, the authors mention that they exclude patents that are filed to individual inventors. Yet, they do not support this decision with any arguments. Likewise, the authors state that they aim to incorporate an organization's history by only including organizations that had at least one patent application before the observation year. In this case, the authors provide a justification for their decision, yet it would be helpful if they could elaborate on why it is important to take an organization's history into account and why they decided for this specific period.

*Thank you for raising this important point. We explain why we exclude individual inventor’s patents by stating that “as our analysis is conducted on organizational level and we focus on organizations and their granted patents, thus patents filed to individual inventor are excluded from the dataset” on Page 18.*

*We elaborate on why it is important to take an organization’s history into account by stating “It is because several measurements in our analysis requires the organization’s patenting history. For example, we rely on organizations’ previous patents to obtain their technology portfolio and calculate technological proximity. We also rely on organizations’ previous patents to calculate accumulated patent counts and obtain the measure of past performance. We started our analysis from the year of 2000 as from this year on, nanotechnology patents increased dramatically. We ended our analysis in 2018 as our patent data is only updated till 2018.”*

5. Statistical analysis

Although the authors mention that they test for potential multicollinearity with the variance inflation factor (VIF), they do not control for any other assumptions necessary to conduct a logistic regression (e.g. independence of observations, no outliers, linear relationship between explanatory variables and the logit of the response variable). To obtain sound results, the authors should check for these assumptions since they could affect their empirical results and thus their theoretical contribution. Additionally, it would be helpful if the authors could include the R-squared value and the F1 score. Otherwise, it is hard to determine how suitable the model is for their data (how much variance in the dependent variable can be explained by the model?). Currently, the authors perform aggregated analyses with their data ranging from 2000-2018. To receive a more dynamic understanding of the relationship between the different variables, it would be interesting to perform longitudinal analyses since the authors work with time-series data.

*We are grateful for your advice. In the revised version, as you suggested, we re-run the regression analysis using the random-effect panel Logit model to have a more dynamic understanding of the relation. The results are displayed in Table 5. We also updated the Descriptive analysis and correlations of variables accordingly in Table 3 and 4.*

*Before the Logit analysis, we perform several tests including the Hausman test, the Durbin-Watson test, the calculation of Cook's Distance of the variables, and OLS regression. These tests can ensure the independence of observations, no outliers in the variables, and the linear relationship between explanatory variables and the logit of the response variable. The description is on Page 26.*

*We included the Wald chi2, Prob>chi2, and Log likelihood of the regression analysis. Prob>chi2 can reflect how much variance in the dependent variable can be explained by the model.*

6. Exemplary issues regarding language and format:

Grammar and wording:

- In a lot of cases, the authors forgot to mention the respective article in front of a noun (e.g. "Then, in the purpose of opening the “black box” of relation between proximity and recombination innovation, we viewed knowledge base as a structural factor during technological adaptation process among organizations."); please check the entire manuscript.

- p.1: "While to date, our comprehension of how partnering organizations access a broader knowledge pool, uncover novel perspectives, and spot recombination opportunities on the technological landscape is limited [6, 7]."; the word "while" needs to be deleted, otherwise one would expect a counter-argument at the end of the sentence.

- p.2: "Notably, existing studies has highlighted the importance of organizations’ external R&D collaboration as a critical mechanism to recombine novel knowledge from partners, the process of which would spur more recombination innovation."; the "has" needs to be changed into "have."

- p.2: I do not understand the following sentence: "Knowledge network represents the coupling logic of organizations and guilds organizations knowledge searching process."; please rewrite this sentence.

- p.2: "Previous studies puts forward that knowledge base is a network of knowledge element in essence [6, 19, 21, 22] that has great implications for formation of collaborations [20, 23], collaborative effectiveness [18, 20], and learning of partners’ knowledge [9]."; "puts" needs to be transformed into "put", "element" needs to be the plural form, "has" needs to be changed to "have", and there is an article missing before formation.

- p.3: "We detailly concentrate on two marked aspects of knowledge network which are knowledge base decomposability i.e., the extent to which knowledge elements are connected with each other forming clustered structure, and degree centrality of knowledge elements i.e., the extent to which knowledge elements are connected to other knowledge elements within and beyond organizational boundary."; to my knowledge, the word "detailly" does not exist, please revise. Additionally, "structure" should be transformed into the plural form.

- p.7: "While, too much similarities in knowledge base would hurt the alternative knowledge that can be considered limiting the recombination scope [47]."; the authors should delete the "while" and "much" needs to be changed to "many."

- p.9: "Therefore, locating in a vicinity of collaborating partners always means a high level of relationship quality, promotes the mutual understanding, and supports creativity and information exchange."; "locating in a vicinity" is hard to understand, simply rewrite it to e.g., "being spatially close to collaborating partners."

- p.20: "It means that large organizations and large research teams do not well perform in the realm of recombination innovation."; please change "well perform" to "perform well."

*We are grateful for your advice. In the revised version, we revised the paper according to your suggestions and corrected the grammar and wording in the paper.*

References

- p.26: "However, previous studies stated that joint patenting can be seen as the result of enduring R&D collaboration between organizations."; please provide some sources when mentioning "previous studies."

*We added two references [66, 67] in this section.*

*[66] Lin C, Wu Y-J, Chang C, et al. The alliance innovation performance of R&D alliances—the absorptive capacity perspective. Technovation 2012; 32: 282–292.*

*[67] Fritsch M, Titze M, Piontek M. Identifying cooperation for innovation―a comparison of data sources. Industry and Innovation 2020; 27: 630–659.*

Format

- It seems like formulas were included with a screenshot and are therefore of low-resolution quality. Please check and insert them directly in the document. If possible, also increase the resolution of the inserted figures.

*Thanks for your suggestions. We re-wrote the formulas in the paper using Word Formula.*

7. Conclusion

In summary, I can state that the authors pursue an interesting line of research that highlights the importance and different effects of proximity on the recombination innovation of companies on a dyad level. Yet, there are some issues, particularly regarding their operationalization of variables and their statistical analyses that could be improved. I hope my suggestions are helpful and wish them success as they continue their research.

*Thank you for your continued support and guidance!*

**Reviewer 2 comments：**

The paper illustrates the connection between proximity of firms and the creation of recombination-based patents at the dyad-level. Proximity of firms is considered through geographic proximity, technological proximity and the similarity of company-level knowledge bases.

I find the basic idea behind the paper interesting, however, I have a list of comments and suggestions for improvement below:

1. The first sentence of the abstract is very hard to follow. I would suggest to please the readers and catch their attention with an easy-to-understand beginning.

*Thank you so much for these comments. We rewrote the first sentence of the abstract.*

2. The applied methodology is not mentioned/hinted in the abstract at all.

*In the abstract, we added the methodology used in the paper.*

3. The Proximity, knowledge base and recombination innovation section of the paper does not refer to the growing literature about atypical combinations and teamwork. I would suggest to build on this stream of literature to strengthen the motivation of the paper. Why are recombination innovation important in general?

Uzzi et al. (2013) Atypical combinations and scientific impact. Science 342 (6157), 468-472.

*Thank you for raising the point. We included this reference in the revised version of the paper.*

*In the introduction section on Page 1 we added:*

*“Studies also show that the atypical combination of conventional knowledge spanning a broader range of technological domains often produces inventions with unusual high impacts [3].”*

4. In general, the paper would benefit from descriptive figures that illustrate the sample and the key concepts. For example, network metrics are applied, so an illustrative example network (or two, since the focus is on dyads) could make the paper more appealing and easier to follow. It would be nice to see the distribution of the number of patents and the number of technology classes by companies in the final sample.

*Thank you for pointing us to this limitation. In the revised version of the paper, we plotted the example knowledge network (illustrating both high and low level of knowledge base decomposability and degree centrality) in Figure 2 and 3 on Page 26.*

*We analysed the distribution of the number of patents and the number of technology classes by companies in the final sample. We show some descriptive indexes in Table 1 on Page 25.*

6. Additionally, illustration on how balanced are dyads in terms of patents by company and IPC classes filed by company would be nice to see. Is it the case that large, productive and often specialized companies tend to introduce new combinations in cooperation with smaller, more innovative, but less experienced firms? In such a situation, a small firm with a single patent would have a highly clustered knowledge network by definition. Clear descriptive on this regard would help.

*We show some descriptive indexes about the distribution of the number of patents and the number of technology classes at the level of collaborative dyads in Table 2 on Page 25. We analysed the indexes on Page 25 as follows:*

*“Table 2 presents the distribution of the number of patents and the number of technology classes of collaborative dyad organizations. As shown, in the subgroup of co-patenting organizations that does not produce recombination innovation, the dyad organizations have the average patent number of 34 and 26 (Technological classes: 14 and 10). Comparably, in the subgroup of co-patenting organizations that produces recombination innovation, the dyad organizations have the average patent number of 13 and 13 (Technological classes: 7 and 20). It shows that, on average, specialized organizations tend to introduce new combinations in cooperation with other similar-sized organizations with diverse sets of technologies.”*

7. The dependent variable (recombination innovation) only considers combinations that are new to the two companies in the focal dyad. Since patents represent highly codified innovation, firms can put resources together to more effectively copy an already existing solution (or moved towards a technological solution). Could you illustrate how often the observed dyads introduce new combinations that are novel for the entire sample of companies? Focusing only on absolute novel combinations might lead to a too small sample size in your context (?), however, discussing your results in relation to other studies highlighting the role of geography for the first combination of technology classes on patents might improve the paper.

Juhász et al. (2020) Explaining the dynamics of relatedness: The role of co-location and complexity. Papers in Regional Science 100 (1), 3-21.

*Thank you for your suggestions. We calculated recombination innovation that is new to the whole sample. This measurement of recombination innovation can capture inventions that contain a higher level of novelty. Our measurement shows that the probability that dyads introduce new combinations that are novel for the entire sample of companies is very low. Among the whole sample of 450 co-patents, only 11 patents that have knowledge combinations that are novel for the entire sample of companies are observed. It would lead to too low probability to conduct regression analysis.*

*As you suggested, we include this reference in our paper and discuss the implication of geography on the first combination of technological classes on Page 31.*

8. The dependent variable (recombination innovation) needs to be described more accurately. Imagine organization A with the patent history of code1, code2 and code3 and organization B with the patent history of code3, code4, and code 5. They create a patent together with code1, code3 and code4. Is this patent a recombination in your definition? It introduces a new technology for both firms that they have not used or combined previously by their own. Is it differentiated in any way from the situation when code1, code2, code3, code4 and code5 are all used in the collaboration-based patent?

*Our definition of recombination innovation includes the case that a new IPC is introduced in the co-patents. We explain the specific measurement of recombination innovation in detail on Page 19 and 20 as follows:*

*“A combination of IPC class is considered new if no patents of the two organizations in the previous years had the same combination of IPC classes. For example, if organization A possess IPC class X and Z. Organization B possess IPC class Y and K. If organization A and B collaborate and create patent 1 with IPC class X, Y and patent 2 with IPC class M, then these two patents are all counted as recombination innovation jointly developed by A and B. It is because patent 1 contains the combination of X and Y that has not appeared in the previous patents of both organization A and B. Patent 2 introduces a new technology M for both firms that they have not used or combined previously by their own. In this way, our measurement of recombination innovation not only includes co-patents that contain a new IPC classification that has not appeared in the previous patents of collaborating organizations, but also the new combinations of existing IPCs.”*

9. I do not think the distance calculation formula is necessary in the main text. Please consider to move it to the SI/appendix.

*Thanks for your suggestions. We moved the distance calculation formula to Appendix 1.*

10. The dyad level independent variables are averages of the respective values. How would the results change in case summarized values would be used alongside controls for absolute differences in regressions?

*Thanks for your suggestions. We added a robustness check section on Page 29. In this analysis, we utilized the alternative measurements of control variables including the sum and difference of values of collaborating organizations’ past patent performance, knowledge variety, age, and the usage of internet. The results of the robustness check analysis are displayed in Table 6. The results are similar to our main results.*

11. Due to the bipartite-to-unipartite network projection of patent-IPC code combinations, the knowledge network of companies will be highly clustered. Could you please show the distribution of the variable? I am suspicious that many companies have a knowledge network that does not show high clustering, but consist of several network components. Is this true? Do companies produce patents in unconnected areas that collaborations make connected? Could you please control for the number of knowledge network components?

*Thanks for pointing out this concern. The mean value of clustering is 0.82 with a minimum value of 0.36 and a maximum value of 1. The measurement shows that most of the organizations have a clustering around 0.3-1. The average clustering is high. There are cases that organizations have very 1-2 patents thus having a clustering of 1. We didn’t exclude these cases. But we found that the exclusion of these cases did not affect our results.*

*As you suggested, in the revised version of the paper, we controlled for the number of knowledge components in an organization’s knowledge network using the variable “Knowledge element”.*

12. A possibly better alternative for the decomposability (clustering) variable would be the ratio of clustering and clustering in a random network with similar size.

*Thanks for your suggestions. We added a robustness check section on Page 29. In this analysis, we utilized the alternative measurements of knowledge base decomposability using the ratio of the clustering coefficient of organizations’ knowledge network and clustering in a random network with the same number of vertices and edges. The results do not support Hypothesis 3. But the sign of the regression coefficient is the same as the regression results in the paper (-7.98). The results support Hypothesis 4.*
