# Supplementary material for: The influence of proximity and knowledge base on recombination innovation in R&D collaboration
Source: PLoS One. 2024 Feb 27;19(2):e0298735. doi: 10.1371/journal.pone.0298735 (PMC10898747; doi:10.1371/journal.pone.0298735)
Supplement: Attachment — Submitted filename: Response letter - round2.docx [file pone.0298735.s003.docx]

**RESPONSE LETTER: PONE-D-22-33904R1**

*Dear editors and reviewers,*

*Thank you for giving us the opportunity to submit a revised draft of our manuscript titled “The influence of proximity and knowledge base on recombination innovation in R&D Collaboration” to Plos One. We appreciate the time and effort you and the reviewers have dedicated to providing your valuable feedback on our manuscript. We are grateful to the reviewers for their insightful comments on our paper.*

*We have been able to incorporate changes to reflect most of the suggestions provided by the reviewers. We have taken your advice seriously and framed the manuscript. Below you can find a point-by-point response to your comments. Again, we thank you wholeheartedly for allowing us to resubmit a minor revision of our study.*

**Reviewer 1 comments：**

1. Unclear research gap

In their introduction, the authors write, "However, despite the recognition of the proximity effect on collaborative innovation, current research fails to throw light upon two essential aspects." It remains unclear what concrete two aspects the authors refer to as they continue the next paragraph with a new topic. The authors could strengthen their argument and identified research gap by naming the two essential aspects directly (which should naturally follow their previous argumentation).

*Thank you for your constructive and useful review. We added a paragraph to briefly discuss (also considering that the length of the introduction cannot be too long) and point out the main two theoretical issues unsolved in the existing studies as follows on Page 2-3.*

*“First, in the existing literature, proximity and collaborative innovation are directly linked in theorization, while the underlying transmission mechanism is, to a large extent, black-boxed [13, 14]. In the economic geography literature, studies have investigated various proximity dimensions, and their effects on the formation and maintenance of collaborations in the context of inter-regional [15] and inventor collaboration [16]. In addition, the effect of proximity on the knowledge sharing and production during inter-organizational alliance is well documented [17]. While in the theorization of the effects of proximity during organization-to-organization collaborations, the role of underlying organizational characteristics remains less salient. Research has rarely examined how proximity and organizational characteristics jointly spur innovation at the collaborative dyad level. Second, existing literature highlights that studies should focus on knowledge space within organizational boundaries when examining inter-organizational collaboration [18]. While, examining the current economic geography literature, few studies have applied the concept of "knowledge space" at the organizational level and link it to the outcomes of cooperation.”*

2. More supporting arguments for deciding on the two aspects of the knowledge network

The authors state in their introduction that they primarily focus on knowledge base decomposability and degree centrality of knowledge elements when evaluating the interaction effects of the knowledge graph on the relationship between technological and geographical proximity on recombination innovation. Unfortunately, the authors do not include any arguments supporting that decision. Why did they decide on these aspects of the knowledge graph and excluded others? The quality of the paper could be improved if the authors could add more arguments for their decision.

*Thank you for your constructive and useful review. As you suggested, we added explanations about why we choose these two types of knowledge base characteristics on Page 4. Mainly, we consider that these two types of knowledge base originated from an organization’s two distinct learning and innovative strategies.*

*“These two dimensions of knowledge base characteristics of an organization can represent two different innovation strategies. Knowledge base decomposability usually originates from an organization’s expertise in specialized fields of knowledge [19, 21]. These organizations often rely on their deep utilization of domain knowledge to maintain existing advantages. The degree centrality of knowledge elements, on the other hand, usually originates from the broader connectedness of technologies within the organization [21, 23]. These organizations tend to rely more on the combinatorial potential of knowledge as well as external knowledge acquisition to exploit the internal technologies fully.”*

3. Potential interaction between technological and geographic proximity

On page 11, the authors write, "The reason lies in that when collaborating agents are located too close, similar technical backgrounds and capabilities result in the draining of knowledge variety and standardization of know-how." I agree that geographical proximity potentially leads to more similar technical backgrounds and capabilities. Yet, this similarity likely impacts the technological similarity of organizations. Thus, I am wondering if both independent variables might be intercorrelated on a conceptual level (as the authors tested for multicollinearity and did not find worrisome results on an empirical level), affecting recombination innovation. Hence, it would be helpful if the authors could include more arguments that sustain the fact that both concepts are independent of each other and, therefore, essential to determine recombination innovation.

*Thank you for raising this important point. In the previous studies on proximity, geographical proximity and technological/cognitive proximity are often independently studied. As you suggested, we added explanations about this issue as follows on Page 10.*

*“Geographic proximity reflects a different dimension of relational characteristic among partnering organizations compared to technological proximity. Technological proximity represents the overlap of technological background and expertise of the organizations, while geographic proximity is the spatial distance between the collaborating organizations. Spatial propinquity encourages frequent face-to-face interactions, thus stimulating the emergence of interpersonal networks across the boundaries of partner organization. This, in turn, increases the opportunities for face-to-face communication of the inventors, facilitating the development of relational trust. Therefore, partnering organizations develop, learn, and adjust over time the idiosyncratic languages needed for the sharing of ‘fine-grained information’ and tacit knowledge. Although organizations located in the closed vicinity of each other also draw on a same set of regional knowledge and tend to share similar values, norms, and “thinking models”, it does not necessarily mean that these organizations have a larger technological proximity. Therefore, geographical proximity and technological proximity are independent from each other and are both essential to determine recombination innovation at the collaborative dyad level. ”*

4. Operationalization of knowledge base decomposability

The authors explain that they measure knowledge base decomposability with the "percentage of closed triads in the sum of closed and open triads in the network according to the formula below." As this formula represents the global/overall clustering coefficient, which the authors also mention later on, I suggest using this naming from the beginning, making clear that "knowledge base decomposability" is a concept that is operationalized through the global/overall clustering coefficient. For more information, please see:

Jackson, M. O. (2008). Social and economic networks (Vol. 3). Princeton: Princeton University Press.

*Thank you for raising this important point. As you suggested we clarify that the knowledge base decomposability is obtained using the global clustering coefficient of the network on Page 21.*

5. Clarifying degree centrality

The authors measure the degree centrality of knowledge elements by summing up the degree centrality of all knowledge elements. I assume the authors correctly apply the normalized degree centrality as they compare values across graphs. It would be helpful if the authors could mention this information within the manuscript.

*We are grateful for your advice. In the revised version, we clarified that we used the normalized degree centrality on Page 22.*

6. Statistical robustness test

I acknowledge the authors' effort of having included several statistical robustness tests for their statistical model. I want to add some minor remarks that could potentially improve the quality of this section. First, as not all readers are familiar with the statistical test applied, please briefly describe each test's purpose (e.g., why did you apply the Hausmann test?). Second, you mention on p. 26 that you calculated the VIF without providing any results. On p. 27, you refer again to the VIF and mention the respective results. Please resolve this redundancy and immediately state the results of the VIF and the potential implications (which are none in your case). Third, I am wondering how you used an OLS to check for a linear relationship within the model. Did you do a visual analysis? It would be great if you could elaborate on your approach in more detail. Fourth, you write that "... the past performance of the organizations is negatively related to the recombination innovation in collaboration." I am not sure what you mean by "performance." It would be great if you could state more explicitly what variables relate to performance.

*Thanks for the suggestion. We state the results of VIF immediately after first stating it on Page 26. As you suggested, we further explain the purpose of Hausmann test, Durbin-Watson test, and Cook’s distant respectively on Page 26. We conduct OLS test before Logit regression to see whether there is leaner relations between the explanatory variables and the logit of the response variable. We use the scatter plot and linear fitting plot to verify the linear relation. The results show that there is a significant linear relation between explanatory variables and the logit of the response variable. We clarify this on Page 27.*

*The past performance is a control variable in our study. It is measured using the number of accumulated patents of the organizations. We explain it clearly on Page 26.*

7. Results of regression with alternative measures

To test the robustness of their model, the authors conduct an additional regression with alternative measures. I think this additional robustness check is a valuable addition to the paper and improves its quality. The authors state that the additional regression yields similar results compared to their base model. To improve the quality of the paper even further, it would be great if the authors could elaborate in more detail if all of the main effects stay the same and, if not, provide some potential explanations for this finding.

*Thanks for the suggestion. The results of the robustness check are same except for one main effect. We added a possible explanation of the result as follows on Page 28.*

*“The results show similar effects of the independent variables except that the moderation effect of decomposability on the relation between technological proximity and recombination innovation is not significant. The sign of the coefficient is same as the results. The possible explanation is that the measurement of global network clustering coefficient comparing to the random network usually has smaller variance as it takes into account network size and density. As a result, the moderation effect of decomposability is less obvious using this alternative measure.”*

8. Minor issues

- Gramma:

p. 0: "We validated the theoretical hypotheses using the Logit regression models based on the longitudinal data of 150 organizations in the global nanotechnology industry."; please delete "the" as it is not necessary in this sentence.

p. 0: "By contrast, the degree centrality of the knowledge element positively moderates the effect of both technological and geographic proximity."; please exchange "By" with "In."

p. 0: "In the last years, recombination innovation has been assuming increasing importance since the miniaturization ... "; please exchange "been assuming" with "gained."

p. 3: "... lays the foundation for an organizations ability to recombine partners’ knowledge... "; please write "organization's."

p. 6: "With the notion of cognitive proximity or technological proximity, actors share the same knowledge base and thus able to..."; please include "are" between "thus" and "able."

p. 6: "on the other hand, social proximity..."; please capitalize "On."

p. 18: "Then, as our analysis is conducted on organizational level and we focus on organizations and their granted patents, thus patents filed to the individual inventors are excluded from the dataset. Our initial set of data consists of a total of 11000 patents."; please delete the "thus" in this sentence.

p.20: "For example, if organization A possess IPC class X and Z. Organization B possess IPC class Y and K."; please combine these two sentences into one and rewrite "possesses."

p.24: "In addition, the level of utilization of theInternet might influence the effect of geographic proximity on collaborative innovation."; please include a space between "the" and "Internet."

p. 24: "Different types of organizations may different innovative patterns ..."; please include "have" between "may" and "different."

*We are grateful for your advice. In the revised version, we revised the paper according to your suggestions and corrected the grammar and wording in the paper.*

- - References

Some of the references contain DOIs, and others do not. Please check and use a consistent citation style.

*We are grateful for your advice. In the revised version, we revised the reference style according to your suggestions.*

9. Conclusion

In summary, I am satisfied with the authors' edits and the current version of the paper. Yet, I raised a few issues that could be addressed to improve the quality of the paper. I hope these suggestions are helpful for the authors and wish them success as they continue their research.

*Thank you for your continued support and guidance!*

**Reviewer 2 comments：**

1. “In addition, in the existing literature, proximity and collaborative innovation are directly linked in theorization, while the underlying transmission mechanism is, to a large extent, black-boxed [13, 14]. “

I would use this sentence to illustrate a possible improvement of this work in terms of positioning. Studies have already used patent data to investigate the role of proximity in innovation related collaboration (co-patenting, co-publication, ...). However, this is often done at the regional level (e.g. Tóth et al. 2021) or at the individual level (e.g. Ter Wal et al. 2013).

I think that your study provides new insights into what drives a firm-to-firm (organization-to-organization) relationship to become an innovation. I think it would help the study if you could articulate this better around the abstract/introduction/discussion. In particular, I would highlight your focus on firm-firm relationships and you are interested in the characteristics of organizations that lead to innovation/patenting.

References:

Tóth, G., Juhász, S., Elekes, Z. And Lengyel, B. (2021) Repeated collaboration of inventors across European regions. European Planning Studies, 29 (12), 2252-2272.

Ter Wal, A. L. J. (2014) The dynamics of the inventor network in German biotechnology: geographic proximity versus triadic closure. Journal of Economic Geography, 14 (3), 589-320.

*Thank you so much for these comments. We rewrote the theoretical contributions of our study in the introduction on Page 2-3 as follows.*

*“First, in the existing literature, proximity and collaborative innovation are directly linked in theorization, while the underlying transmission mechanism is, to a large extent, black-boxed [13, 14]. In the economic geography literature, studies have investigated various proximity dimensions, and their effects on the formation and maintenance of collaborations in the context of inter-regional [15] and inventor collaboration [16]. In addition, the effect of proximity on the knowledge sharing and production during inter-organizational alliance is well documented [17]. While in the theorization of the effects of proximity during organization-to-organization collaborations, the role of underlying organizational characteristics remains less salient. Research has rarely examined how proximity and organizational characteristics jointly spur innovation at the collaborative dyad level. Second, existing literature highlights that studies should focus on knowledge space within organizational boundaries when examining inter-organizational collaboration [18]. While, examining the current economic geography literature, few studies have applied the concept of "knowledge space" at the organizational level and link it to the outcomes of cooperation.*

*In order to fill these gaps, we focus on organization-to-organization collaboration to further contribute to strengthening this body of work on the collaborative nature of innovation and the role of proximity in this process. ”*

*We also added more clarification about the theoretical contribution in the abstract on Page 0 and discussion part on Page 31. We highlighted that our contribution is mainly in the filed of economic geography and recombinant search literature to better position the paper.*

2. In economic geography and related literature, "knowledge networks" are seen as networks of (formal/informal) knowledge exchange, and what you call in your paper a knowledge network is more properly called a "knowledge space".

With respect to knowledge networks, the impact of cognitive/technological proximity on the formation and maintenance of collaborations is well documented.

However, the application of the idea of "knowledge space" at the firm/organisational level and linking it to the outcomes of cooperation is (to my knowledge) new in the economic geography literature. I would suggest that you make your contribution clear and possibly position your setting relative to their previous findings.

Useful references for knowledge network evolution:

Balland, P. A., Belso-Martinez, J. A. and Morrison, A. (2016) The Dynamics of Technical and Business Knowledge Networks in Industrial Clusters: Embeddedness, Status, or Proximity? Economic Geography, 92 (1), 35-60.

Juhász, S. And Lengyel, B. (2018) Creation and persistence of ties in cluster knowledge networks. Journal of Economic Geography, 18 (6), 1203-1226.

Usefull references for knowledge space:

Balland, P. A., Rigby, D. and Boschma, R. (2015) The technological resilience of US cities. Cambridge Journal of Regions, Economy and Society, 8, 167-184.

Hidalgo, C. A. (2021) Economic complexity theory and applications. Nature Reviews Physics, 3 (2), 92-113.

*Thanks for your suggestion. We replace the knowledge network with the “knowledge space” in the revised version of the paper. We clarify on Page 3 and Page 22 that the knowledge space is constructed using the interdependence network formed among different knowledge elements (IPC). We also illustrate the theoretical contribution of the paper regarding the conceptualization of “knowledge space” on Page 34 as follows.*

*“Shifting the conceptual lens from technological components to the interdependence or structure of knowledge elements, we apply the idea of "knowledge space" at the organizational level and link it to the outcomes of cooperation.”*

3. Following the example of recombination innovation on page 19. I argue that patent 2 with the new M technology is not a recombination innovation. Neither of the two companies have used this technology before, so they are not "recombining" their knowledge, but collaborating to learn something that is completely new to both of them.

How would your results change if you would remove cases of such recombination innovation, where we actually observe a "new to both" (and in some cases "only new codes to both") combinations?

*Thank you for your suggestions. For a robustness check, we remove such cases when collaborative organizations create a parent that contains an IPC code that is new to both of them. The cases are relatively small only accounting for 10% of the whole sample. We run the regression with the alternative measure of recombination innovation and obtain similar results. We add this in the Robustness check section on Page 28 as follows.*

*“We also utilized an alternative measure of recombination innovation. When collaborative organizations create a patent that contains IPC codes that is new to both of them, they are collaborating to learn something that is completely new to them. We remove cases of such recombination innovation to obtain an alternative measure. For example, if organization A possesses IPC class X and Z and organization B possesses IPC class Y and K. If organization A and B collaborate and create patent 1 with IPC class X, Y and patent 2 with IPC class M, then patent 1 is considered recombination innovation while patent 2 is not. In the regression results with the alternative measure of recombination innovation all of the main effects stay the same.”*
